# Functional Assembly of Protein Fragments Induced by Spatial Confinement

**DOI:** 10.1371/journal.pone.0122101

**Published:** 2015-04-15

**Authors:** Yongsheng Yu, Jianpeng Wang, Jiahui Liu, Daishun Ling, Jiang Xia

**Affiliations:** 1 Department of Chemistry, Center of Novel Biomaterials, The Chinese University of Hong Kong, Shatin, Hong Kong, China; 2 Institute of Pharmaceutics, College of Pharmaceutical Sciences, Zhejiang University, 866 Yuhangtang Road, Hangzhou, Zhejiang 310058, China; Russian Academy of Sciences, Institute for Biological Instrumentation, RUSSIAN FEDERATION

## Abstract

Natural proteins are often confined within their local microenvironments, such as three-dimensional confinement in organelles or two-dimensional confinement in lipid rafts on cytoplasmic membrane. Spatial confinement restricts proteins' entropic freedom, forces their lateral interaction, and induces new properties that the same proteins lack at the soluble state. So far, the phenomenon of environment-induced protein functional alteration still lacks a full illustration. We demonstrate here that engineered protein fragments, although being non-functional in solution, can be re-assembled within the nanometer space to give the full activity of the whole protein. Specific interaction between hexahistidine-tag (His-tag) and NiO surface immobilizes protein fragments on NiO nanoparticles to form a self-assembled protein "corona" on the particles inside the nanopores of mesoporous silica. Site-specific assembly forces a shoulder-by-shoulder orientation and promotes fragment−fragment interaction; this interaction together with spatial confinement of the mesopores results in functional re-assembly of the protein half fragments. To our surprise, a single half fragment of luciferase (non-catalytic in solution) exhibited luciferase activity when immobilized on NiO in the mesopores, in the absence of the complimentary half. This shows for the first time that spatial confinement can induce the folding of a half fragment, reconstitute the enzyme active site, and re-gain the catalytic capability of the whole protein. Our work thereby highlights the under-documented notion that aside from the chemical composition such as primary sequence, physical environment of a protein also determines its function.

## Introduction

As the essential biocatalysts for chemical conversion in life, enzymes are very sensitive to their physical environment. The crowding effect represents one of the environmental aspects chemists have explored. The cytoplasmic space of a cell is crowded by a variety of macromolecules with a total concentration approaching 40% [1–4]. The crowding environment, in contrast to that of diluted aqueous solution in test tube, can significantly alter the internal enzyme dynamics as well as catalytic activity [5]. For example, Guo and co-workers revealed that macromolecular crowding could change the structure and increase the intrinsic catalytic efficiency of the isochorismate synthase, and even suppress the side products [6–8]. Spatial confinement represents another environmental factor that affects the property of biomacromolecules [9–11]. For example, immune synapse comprises many proteins within a two-dimensional space at cell-cell junction [12]. Also chaperonin confinement helps to smooth folding landscape and to promote protein folding [13]. Surface-immobilization of proteins and protein encapsulation in porous materials have been used to mimic spatial confinement in life [14, 15]. Spatial confinement in nanopores has been shown to stabilize α-helix structure, and destabilizes β-structures [16]. This effect of environment—induced structural transition has been proven in multiple proteins [17–20]. Pande and co-workers recently revealed that confining protein alone promoted protein folding but confining both protein and solvent resulted in unfolding [21]. Wolynes and co-workers showed that excluded volume effects due to spatial confinement resulted in correlations between molecules even without specific interactions, which affected the kinetics and thermodynamics of protein—protein interaction [22]. The majority of these studies relied on theoretical simulation of the folding states of the protein. Experimental illustration on environment—induced gain of function has been scarce. On the other hand, entrapment of enzymes in solid supports, such as mesoporous materials has been amply studied owing to the long-standing industrial use of immobilized enzymes in biocatalysis. The focus of enzyme entrapment in mesoporous materials however has been the maintenance, enhancement, or variation of the enzymatic activity, as well as the stability and reusability of the biocatalyst [23–25]. It has also been well studied that the proteins can form “coronas” on the exterior of nanoparticles, which provide the biological identity of the nanosized materials [26–30]. This notion has inspired us to consider whether the formation of the corona in turn grant the protein new functions.

**Fig 1:**
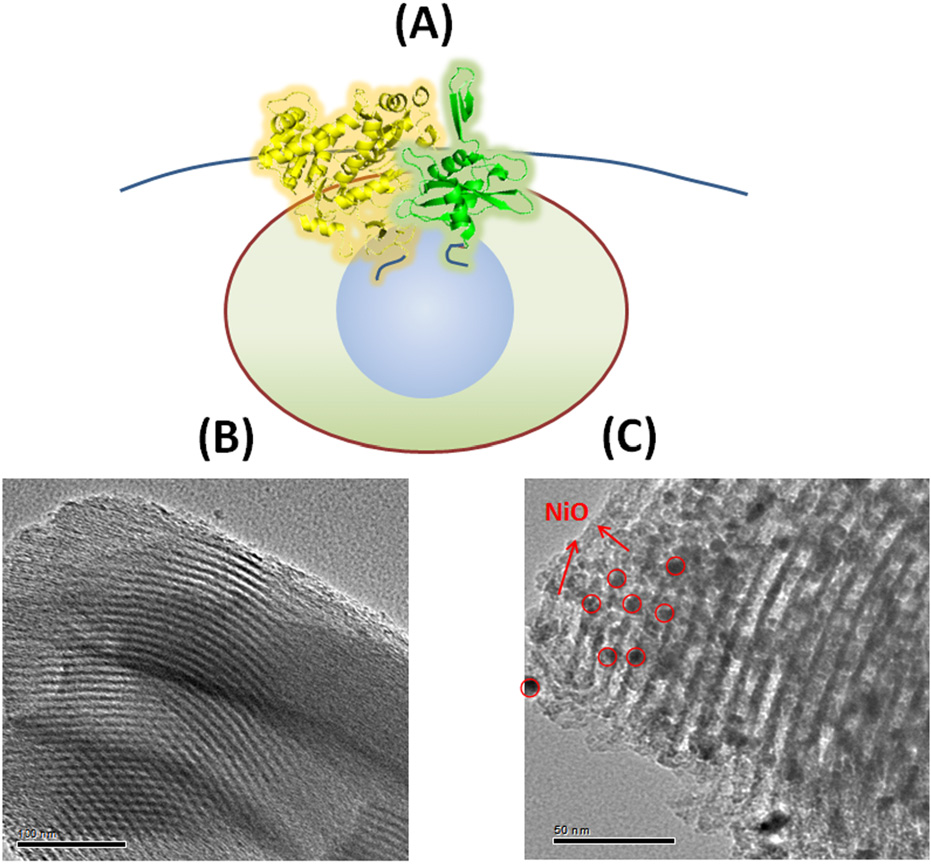
SBA-NiO, a mesoporous silica material decorated with NiO nanoparticles, as the scaffolding support for protein fragments to interact. (A) Schematic illustration of the interaction of protein half fragments induced by site-specific interaction of His-tags with NiO nanoparticles in the cavities of mesoporous SBA material. (B) TEM image of as synthesized SBA-15. (C) TEM image of SBA-NiO.

In a recent report, we described the design and synthesis of NiO nanoparticle-decorated mesoporous silica (SBA-NiO) and demonstrated that hexahistidine-tagged (His-tagged) enzymes can be stably immobilized in the mesopores through His-tag—NiO interaction [23]. The immobilized enzymes showed activity increase towards substrate catalysis, and exhibited high durability in multiple rounds of catalysis. Inspired by the success of SBA-NiO in enzyme immobilization, here we seek a different use of the same material (Fig 1A). The mesopores of SBA-NiO can imitate the microenvironment of biomolecules in nature. In particular, site-specific immobilization through the terminal His-tag ensures that the immobilized proteins adopt a uniform orientation. Spatial confinement (both two-dimensional confinement on the surface of NiO nanoparticles and three-dimensional confinement within the mesopores) of biomolecules of a uniform orientation can thereby force lateral interaction between protein molecules. In contrast, immobilization driven by non-specific interaction between the material and the surface of proteins will result in random protein—protein interaction. We demonstrate here that confining meticulously designed half fragments of proteins on the NiO nanoparticles in the mesopores induces new interaction modes between protein fragments, and results in re-gain of fluorescence property and enzymatic activity.

## Materials and Methods

### Plasmid construction

Synthetic DNA oligonucleotides were purchased from Invitrogen (Hong Kong). Gel extraction kits, restriction endonucleases and plasmid miniprep kits were all purchased from Takara (Hong Kong). Sequencing reactions were conducted at BGI (Hong Kong). Bovine Serum Albumin (BSA) was purchased from Life Technologies (Hong Kong). The DNA fragments coding for the four half fragments of mCherry (mCherry_1–159_, mCherry_1–136,_ mCherry_160–237_ and mCherry_137–237_) were amplified from the vector pQL81-mCherry. A nucleotide sequence encoding His-tag was ligated to either N or C terminus to give four DNA fragments N1-mCherry (mCherry_1–159_, with an N terminal His-tag), N2-mCherry (mCherry_1–136_, with an N terminal His-tag), and two C terminal fragments C1-mCherry (mCherry_160–237_, with a C terminal His-tag) and C2-mCherry (mCherry_137–237_, with a C terminal His-tag). A gene fragment encoding a peptide spacer, SSGSSGLVPRGSSGKLAAALE, was inserted between mCherry_160–237_ and His-tag, and between mCherry_137–237_ and His-tag, so that when the N and C half fragments were immobilized on the surface, they are correctly aligned. The DNA encoding N1-mCherry, N2-mCherry, C1-mCherry and C2-mCherry were then inserted into *BamH* I and *Xho* I sites of pGEX-6p-3 to yield plasmids N1-mCherry, N2-mCherry, C1-mCherry and C2-mCherry (S1 Fig). The DNA fragments encoding two half fragments of firefly luciferase (Luc_2–416_ and Luc_398–550_) were amplified from a plasmid encoding firefly luciferase. A nucleotide sequence encoding His-tag was ligated to the N terminus of Luc_2–416_ or the C terminus of Luc_398–550_ to give N-Luc (Luc_2–416_, with an N terminal His-tag) and the C terminal fragment C-Luc (Luc_398–550_, with a C terminal His-tag). The DNA encoding N-Luc was then inserted into *Hind* III and *Xho* I sites of pGEX-6p-3 and C-Luc was inserted into *EcoR* I and *Hind* III sites of pGEX-6p-3 to give expression plasmids N-Luc and C-Luc (S2 Fig).

### Protein expression and purification

Plasmids were transformed into *E*. *coli* BL21 (DE3) cells and colonies were grown overnight at 37°C in LB media supplemented with 100 μg/mL ampicillin. The starter culture was grown overnight, and then was used to inoculate 600 mL LB media supplemented with antibiotics. The cell culture was grown at 37°C to reach OD_600_~0.6 before 1 mM IPTG was added to induce protein expression. After grown at 16°C overnight, cells were harvested and resuspended in 30 mL of lysis buffer (containing 20 mM tris, 500 mM NaCl, 3 mM DTT, 0.1 mM PMSF, pH 7.5), sonicated, and centrifugated at 20,000 g for 1 hour to obtain the supernatant and remove cell debris. GST tagged proteins were allowed to bind to the pre-equilibrated glutathione-agarose resin (GE healthcare, HK) for 45 min. GST tag was then removed by PreScission protease on resin and the protein fragments were eluted, ultrafiltered and stored in TGE buffer (50 mM Tris, 0.5 mM EDTA, 50 mM NaCl, 5% glycerol, pH 7.5) at 4°C. The purity of the proteins was confirmed by SDS-PAGE. The fluorescent spectra of mCherry fragments in solution were measured on a Hitachi F-7000 fluorescence spectrophotometer in a 0.5 mL quartz cuvette in TGE buffer (protein concentration: 1mg/mL) with an optical path length of 1 cm. The excitation wavelength was set at 587 nm and the fluorescent emission was monitored from 580 nm to 780 nm.

### Synthesis of SBA-NiO

SBA-NiO was synthesized following the reported protocol [23]. Briefly, pluronic P123 (2.0 g) was dissolved in water and 2 M HCl at 35°C, followed by the addition of tetraethyl orthosilicate (4.4 g). The mixture was stirred at 35°C for 24 h, and then aged at 100°C overnight without stirring to allow solid product to form. The solid product was washed, air-dried, and calcinated by slowly increasing the temperature from room temperature to 500°C over 8 h and keeping at 500°C for 6 h to yield while powders of SBA-15. SBA-NiO was then prepared by wet impregnation. In a typical procedure, an aqueous solution of NiCl_2_ (0.1g, 1.1 wt %) was impregnated with 1 g of dry SBA carrier, stirred overnight at room temperature and centrifuged. The material was dried and heated to 500°C in air for 5 h to allow Ni^2+^ salts to be oxidized to form ultrafine NiO nanoparticles, which finally gave SBA-NiO.

### Fragment re-assembly on SBA-NiO and fluorescence measurement

The protein fragments, N1-mCherry, or C1-mCherry, or an equimolar mixture of two fragments (1.8 mg/mL each, total 30 μL) were added to the solid powder SBA-NiO and incubated at 4°C for 2 h. 20 μL of the mixture was then dropped on a glass cover-slip and observed under an inverted fluorescence microscope (IX71, Olympus, Tokyo, Japan) equipped with a 100 W mercury lamp (OSRAM HBO 103w/2 Mercury Arc Lamp, Osram Inc., Berlin, Germany), with the excitation wavelength set to be 587 nm. The fluorescent pictures were recorded. The emission spectrum of the fluorescent signal was recorded by a fiber optic spectrometer (QE65000, Ocean Optics, Dunedin, FL, USA). Quantitative measurement of the fluorescent signal was conducted by capturing the fluorescent pictures of the mCherry/SBA-NiO droplet on coverslip by a confocal fluorescent microscope (Nikon C1, Japan). For each picture, at least 10 random areas of the SBA-NiO pictures were selected, and the total fluorescent intensity within the selected areas was measured by EZ-C1 3.90 FreeViewer software (Nikon, Japan), and quantified as were chosen, the relative intensity was calculated as Intensity per Area (a.u./μm^2^).

### Luciferase activity measurement

N-Luc and C-Luc fragments were assembled with SBA-NiO following the same protocol as mCherry fragments. Briefly, 0.5 mg/mL of each fragment (total 30 μL) was incubated with SBA-NiO. 20 μL of slurries or solutions of the protein/SBA-NiO solutions were placed in 96 well plates, and to each well 100 μL of the substrate was added (Luciferase Assay System, Promega, Wisconsin, USA). The luminescence was measured by a luminometer plate reader (GloMax 96 Microplate Luminometer, Promega, Wisconsin, USA) following the standard luminescence protocol.

## Results and Discussions

### Design, engineering and expression of mCherry half fragments

The synthesis and characterization of NiO nanoparticle-decorated mesoporous silica (SBA-NiO) has been described in our previous report [23]. Briefly, mesoporous silica, SBA-15, was first synthesized by using P123 as a surfactant (Fig 1B) and then NiO nanoparticles with ultrafine structure and uniform size were generated inside the nano-sized pores, the combination of which gives SBA-NiO. X-ray diffraction measurement indicated a face-centered-cubic crystalline NiO structure (2*θ* = 37.27, 43.30, 62.90, 75.44, and 79.43° can be indexed to (111), (200), (220), (311), and (222), respectively) [23]. The Brunauer–Emmett–Teller (BET) surface area was measured to be 680 m^2^ g^-1^ [23]. The pore diameter was measured to be around 8 nm, which can perfectly accommodate several protein molecules. Numerous NiO nanoparticles can be found in the nanochannels under TEM (Fig 1C).

**Fig 2:**
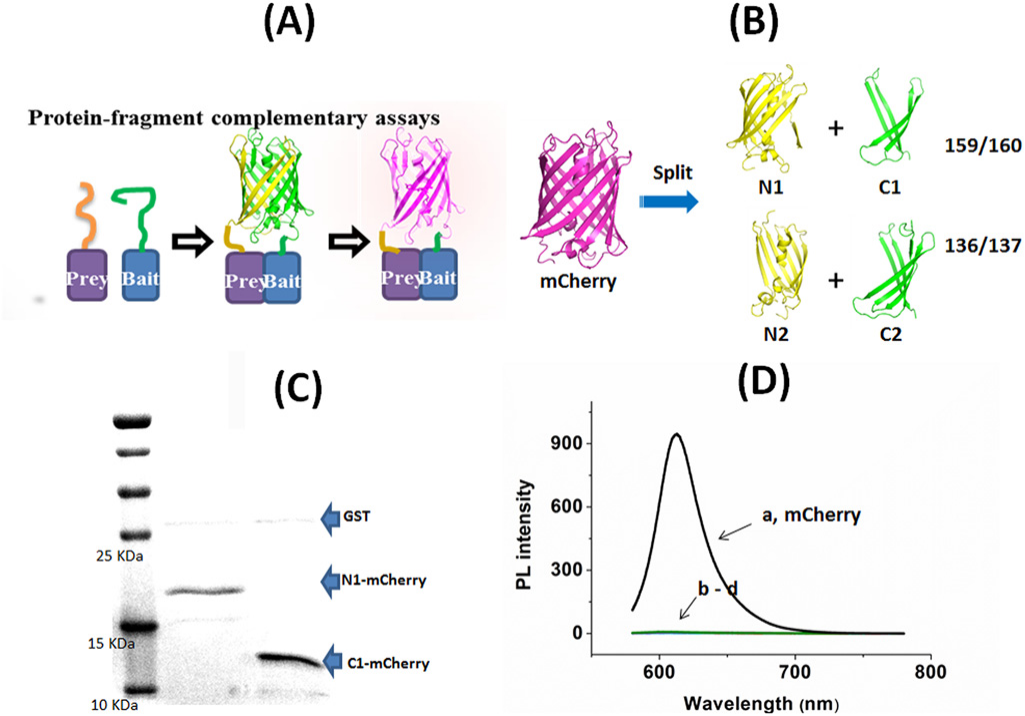
Split fragments of mCherry. (A) Schematic illustration of protein-fragment complementary assay (PCA) using mCherry as a “reporter” protein. (B) Split expression of mCherry at 159–160 position and at 136–137 position give two pairs of fragments N1/C1 and N2/C2 respectively (the structure of mCherry is drawn based on PDB ID 2H5Q) [37]. (C) SDS-PAGE picture of N1-mCherry and C1-mCherry. (D) Normalized photoluminescence (PL) spectra of mCherry (a), N1-mCherry (b), C1-mCherry (c), N1-mCherry + C1-mCherry (d) in solution at 1 mg/ml (λ_ex_ = 587 nm).

To illustrate that SBA-NiO can be used as a structural mimic for spatial confinement of biomolecules, we adopted the engineering strategy of protein-fragment complementary assays (PCAs), a method widely used to identify protein—protein interactions in biological systems [31–33]. In a typical PCA, a “reporter” protein, with well-defined structure and function, is split into two halves (N- and C- termini) and fused with two other domains, a “bait” and a “prey” respectively. Binding of “bait” and “prey” brings the two halves into close proximity with the right orientation to allow for structural reconstitution and functional re-gain of the reporter protein (Fig 2A). We chose mCherry protein, a second-generation variant of the monomeric red fluorescent protein mRFP1 because of its high fluorescence intensity, long excitation and emission wavelength (587 and 610 nm respectively) and shorter maturation time as compared with other fluorescent proteins [34,35]. These properties render mCherry protein the perfect “reporter” protein for bimolecular fluorescence complementation assay. The complementation properties of different mCherry split patterns have been extensively explored by Zhang *et al* [36]. Furthermore, the N terminus and the C terminus of mCherry are very close in space, allowing the both ends to be the anchoring points for protein immobilization (Fig 2B). Splitting the red fluorescent protein, mCherry between amino acids Asp159 and Gly160 resulted in a *complementary* PCA pair, mCherry_1–159_ and mCherry_160–237_, meaning that when they are brought into close proximity by bait—prey interaction, the fluorescence of mCherry protein will be observed. On the other hand, fragments mCherry_1–136_ and mCherry_137–237_ are *non-complementary*, because they cannot be re-constituted to give fluorescent protein [36, 37]. From the crystal structure of mCherry (PDB ID 2H5Q), the 159/160 split site (ED|GA) and 136/137 site (PS|DG) locate at the top and bottom loops of the β barrel respectively (Fig 2B). These two loop regains are highly flexible, so splitting at these two sites is expected to be topologically feasible for the protein assembly [36].

We cloned, expressed and purified the four fragments, two N terminal fragments N1-mCherry (mCherry_1–159_), N2-mCherry (mCherry_1–136_), and two C terminal fragments C1-mCherry (mCherry_160–237_) and C2-mCherry (mCherry_137–237_) (Fig 2B). Hexahistidine tags (His-tags) were appended at the N terminus of N1-mCherry and N2-mCherry, as well as the C terminal ends of C1-mCherry and C2-mCherry. Because the N terminal end of mCherry is slightly longer than the C terminal end, to avoid spatial dislocation and to maximize shoulder-by-shoulder alignment of the N and C half fragments, we inserted a peptide sequence, SSGSSGLVPRGSSGKLAAALE, between C terminal end and His-tag as a spacer. The four fragments were over-expressed as glutathione s-transferase (GST) fusions in *E*. *Coli*, with a proteolytically cleavable linker LEVLFQ|GP between GST and the mCherry fragments. GST fusion apparently helped the overexpression of the half fragments in *E*. *Coli*, because the fragments failed to overexpress without the presence of GST tag. Proteolytic cleavage by the PreScission protease at the linker followed by subsequent purification site-specifically removed the GST tag and yielded the His-tagged proteins according to SDS-PAGE analysis (Fig 2C). Expectedly, the mixture of N1-mCherry and C1-mCherry, or any combinations of the four fragments, or each fragment alone in solution could not generate appreciable fluorescence at 610 nm, the maximum emission wavelength of full mCherry at λ_ex_ of 587 nm. This indicates that there is no specific interaction between the mCherry fragments, so the fragments cannot re-assemble to functional entity in solution (Fig 2D).

### Immobilization of mCherry fragments on SBA-NiO induced functional assembly

**Fig 3:**
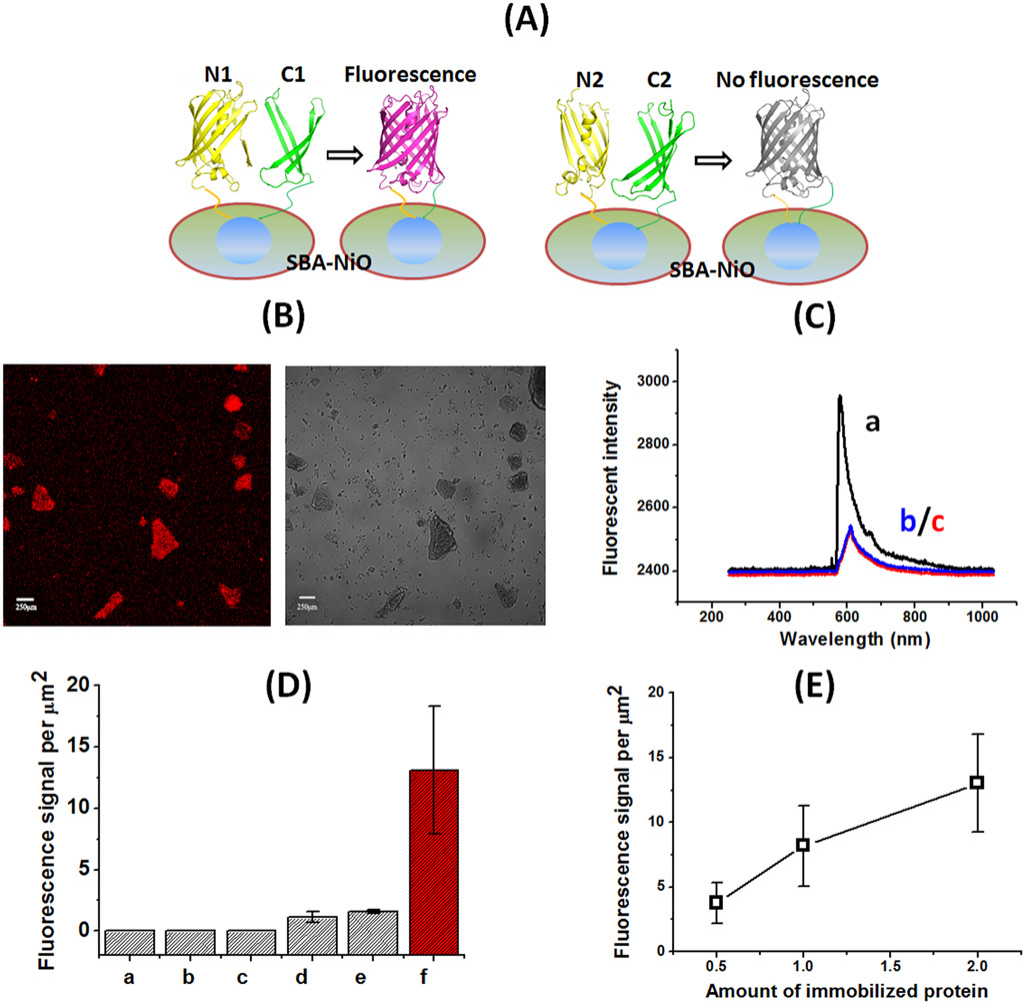
mCherry fragments assembled on SBA-NiO to form fluorescent protein. (A) Schematic illustration of the assembly of mCherry fragments on the surface of SBA-NiO. (B) Fluorescent microscope image of N1-mCherry/C1-mCherry/SBA-NiO (left, TRITC channel; right, bright field). (C) Normalized fluorescent spectra of N1-mCherry/C1-mCherry/SBA-NiO (a), N1-mCherry/SBA-NiO (b), and N1-mCherry/N2-mCherry/SBA-NiO (c) (λ_ex_ = 587 nm). All other assemblies do not fluorescence. (D) Quantification of PL intensity per μm^2^. a, SBA-NiO; b, BSA/SBA-NiO; c, N2-mCherry/C2-mCherry/SBA-NiO; d, N1-mCherry/N2-mCherry/SBA-NiO; e, N1-mCherry/SBA-NiO; f, N1-mCherry/C1-mCherry/SBA-NiO. (E) The PL intensity per area of N1-mCherry/C1-mCherry/SBA-NiO correlated with the amount of immobilized N1-mCherry and C1-mCherry.

We next assembled the mCherry fragments with SBA-NiO through His-tag. Among all the combinations only equimolar combination of N1-mCherry and C1-mCherry on SBA-NiO emitted clear red fluorescence under fluorescent microscope; none of the other combinations, including N2-mCherry/C2-mCherry/SBA-NiO and N1-mCherry/N2-mCherry/SBA-NiO fluoresced (Fig 3). The spectrum of fluorescent pixels from N1-mCherry/C1-mCherry/SBA-NiO showed a maximal emission around 607 nm (λ_ex_ = 543 nm), consistent with the fluorescent spectrum of the folded whole mCherry protein (trace a in Fig 3C) [37]. This indicates that simultaneous immobilization of N1-mCherry (mCherry_1–159_) and C1-mCherry (mCherry_160–237_) in the pores of SBA-NiO promoted spatial proximity between the two fragments in correct orientation, and induced their re-assembly into a functional “reporter” protein which is presumably correctly folded. We further quantified the fluorescent signal by measuring the number of pixels per area of μm^2^ from the fluorescent images taken under confocal microscope. We clearly observed that only N1-mCherry/C1-mCherry/SBA-NiO showed fluorescent signal well above the background, whereas N1-mCherry/SBA-NiO and N1-mCherry/N2-mCherry/SBA-NiO showed only weak signal, less than 10% of that of N1-mCherry/C1-mCherry/SBA-NiO (Fig 3D). As N1-mCherry (1–159) contains the three residues responsible for the formation of the fluorophore (66–68), it then is possible that N1-mCherry can assemble on the surface of NiO nanoparticle to form a hydrophobic environment and allowed the fluorophore to partially form, despite to a much less degree than N1-mCherry/C1-mCherry/SBA-NiO. In a dose-dependent experiment, increasing the amount of N1-mCherry/C1-mCherry on SBA-NiO corresponded with the increase of fluorescent signal, showing that the NiO binding sites have not been saturated under this condition (Fig 3E). This suggests that the binding of N1-mCherry/C1-mCherry to SBA-NiO is not through non-specific adsorption.

### Design, engineering and expression of luciferase fragments

**Fig 4:**
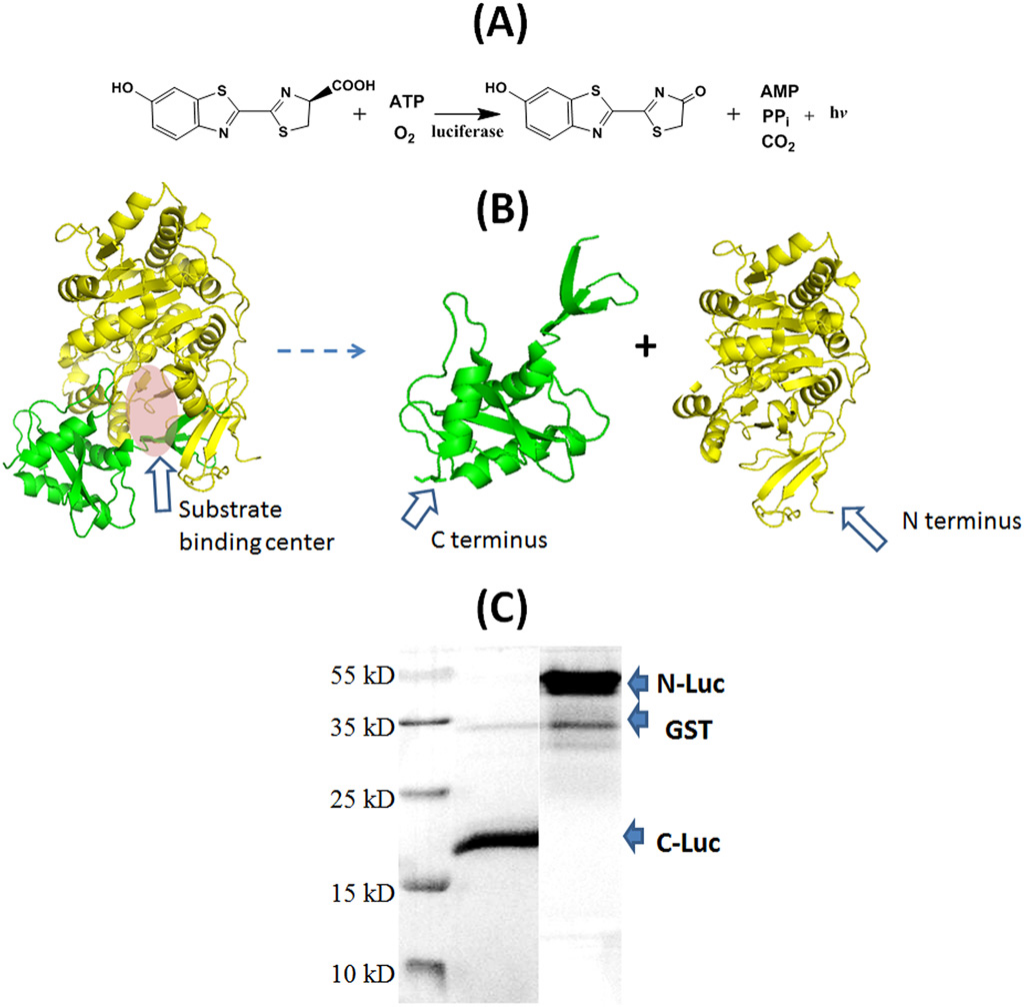
Split fragments of luciferase. (A) Luciferase assay. (B) Split luciferase to give two complementary fragments N-Luc 2–416 and C-Luc 398–550, each his-tagged at N terminus and C terminus respectively (the structure is from PDB ID 2D1S) [39]. (C) SDS-PAGE picture of N-Luc and C-Luc.

**Fig 5:**
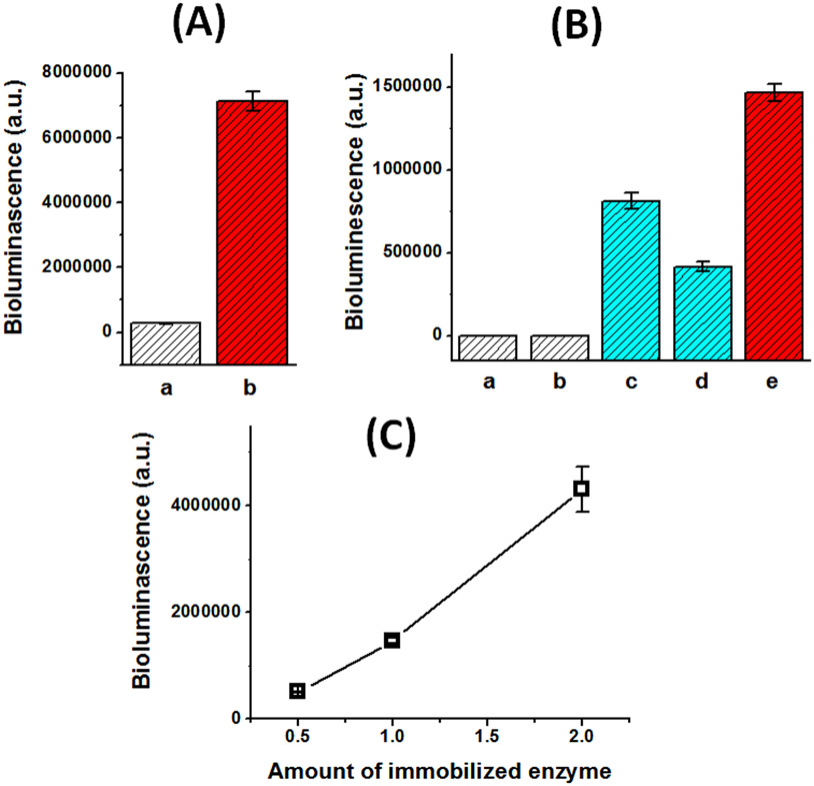
Fragments of Luciferase assembled on SBA-NiO to form functional luciferase. (A) The assembly N-Luc/C-Luc/SBA-NiO catalyzed luciferin conversion, whereas N-Luc + C-Luc in solution remained inactive. a, N-Luc + C-Luc in solution; b, N-Luc/C-Luc/SBA-NiO. (B) Luciferase fragments alone on SBA-NiO N-Luc/SBA-NiO and C-Luc/SBA-NiO can regenerate the enzymatic activity. a, SBA-NiO; b, BSA/SBA-NiO; c, N-Luc/SBA-NiO; d, C-Luc/SBA-NiO; e, N-Luc/C-Luc/SBA-NiO. (C) Luciferase activity of N-Luc/C-Luc/SBA-NiO correlated with the amount of immobilized N-Luc and C-Luc.

We then examined the re-constitution of an enzyme, firefly luciferase. The active site residues of luciferase form a cavity to host and bring together luciferin, ATP and molecule oxygen together in appropriate spatial arrangement to result in oxidation of luciferin to oxy-luciferin, with the concomitant release of AMP, PPi, CO_2_ and the emission of light (Fig 4A) [38,39]. In other words, the residues of luciferase are not directly involved in the chemical reaction, other than forming the substrate-binding pocket. Similar as fluorescent proteins, firefly luciferase can be split into two complementary halves [40, 41]. This splitting site resides at the substrate binding site and thereby disrupts its capability of forming the substrate-binding pocket (Fig 4B). We cloned, expressed and purified the two fragments, the N terminal fragment N-Luc (Luc_2–416_) and the C terminal fragment C-Luc (Luc_398–550_). His-tags were appended at the N terminus of N-Luc and at the C terminal end of C-Luc respectively (Fig 4B). The fragments were also expressed as GST fusions and the GST tags were removed by site-specific proteolysis (Fig 4C). Expectedly, the mixture of N-Luc and C-Luc as well as the fragments alone in solution did not exhibit any appreciable enzymatic activity in conversion of luciferin to oxy-luciferin, monitored by the chemiluminescence associated with this reaction (Fig 5A). Therefore, the fragments do not have internal interaction with each other that is sufficient to result in re-constitution of the enzymatic activity.

### Immobilization of luciferase fragments in SBA-NiO induced functional assembly

We then assembled N-Luc/C-Luc/SBA-NiO by incubating equal amount of N-Luc and C-Luc fragments with SBA-NiO. N-Luc/C-Luc/SBA-NiO hybrid assembly effectively catalyzed the luciferin reaction, shown as drastic enhancement of the bioluminescence signal (Fig 5A). As negative control, SBA-NiO and BSA/SBA-NiO hybrid assembly did not exhibit luciferase activity (Fig 5B). The enzymatic activity also clearly correlated with the amount of N-Luc/C-Luc/SBA-NiO hybrid assembly as increasing the amount of the assembly corresponded with increasing luciferase activity (Fig 5C).

To our surprise, N-Luc/SBA-NiO hybrid assembly, although having only half of luciferase, also exhibited clear luciferase activity, ~55% of N-Luc/C-Luc/SBA-NiO hybrid. Similarly, C-Luc/SBA-NiO accounted for ~29% of N-Luc/C-Luc/SBA-NiO material (Fig 5B). This result was even more striking as C-Luc contains only 152 residues out of a total of 550 residues of the full size luciferase. This unexpected observation showed that spatial confinement can result in new interaction modes between protein fragments in the nano cavity, and new function was resulted from the new interaction mode. It has been reported that Arg 218 serves as the active site residue for luciferin as alteration of this amino acid significantly decreases the affinity of luciferease towards the substrate luciferin, exhibiting a 15–20 fold increase in the *K*_m_ values for luciferin [42]. Notwithstanding the solid support from site-specific mutagenesis, this conclusion applies to enzymatic reaction in solution. Our experiment of immobilized luciferase highlights the difference between soluble and well-folded enzyme in solution and surface-immobilized enzyme. The active center of luciferase was likely partially re-constituted inside the nano pores by assembling the N and C halves. Taken together, spatially confined protein fragments in nanopores attained properties that do not exist in diluted aqueous solution. The reason why N-Luc/SBA-NiO and C-Luc/SBA-NiO can exhibit luciferase activity despite the missing of the other half is still elusive. Notably the action of luciferase does not require the direct involvement of its active site residues in the oxidation reaction; instead, a binding pocket that correctly aligns the reactants luciferin, oxygen and ATP is sufficient. Therefore, a possible explanation is that in N-Luc/SBA-NiO, such a binding pocket is re-constituted due to spatial confinement of N-Luc in the nanopores even in the absence of C-Luc in the assembly; the N-Luc fragment may complement the missing subdomains with the help of the other N-luc fragment at the surface of SBA-NiO, and similarly but to a lesser extent in C-Luc/SBA-NiO. The conventional concept of enzymatic active site however is not capable of fully explaining the regain of immobilized luciferase fragments on the surface. The exquisite elucidation of the exact mechanism of surface-immobilized enzymatic reaction awaits the invention of new powerful physiochemical techniques that can probe the surface biochemistry at molecular or even atomic level.

## Conclusions

It is a well-accepted notion that the chemical composition namely the primary sequence and the three-dimensional folding determine the activity of a protein. However, recent investigations indicated that it is not a complete picture: the physical environment at which a protein resides poses strong influence to its function and activity. In other words, a protein in diluted solution in the test tube might be markedly different from its native state or environmentally constrained states. Chemists have revealed, for one example, that crowding will leads to noticeable alteration in the property of enzymes, raising the concern that enzymes in cytoplasm might behave different from those in test tube. Confinement within a two-dimensional or three-dimensional space will also alter the property of proteins. For example, immobilization on solid support often leads to protein unfolding or activity decrease, due to protein-surface interaction. Rarely, one will observe activity enhancement, let alone gain of new activity. We recently developed a new type of material, SBA-NiO, which includes NiO nanoparticles inside mesopores. The NiO particles will specifically bind with His-tagged proteins, providing a two-dimensional constraint; the mesopores can perfectly accommodate the proteins and provide a three-dimensional microenvironment to further promote protein—protein interaction within confined space. We then examined whether such confinement could yield function or activity the soluble proteins do not have. Through thoughtful protein design, we engineered half fragments of mCherry and luciferase, and proved that complementary fragments of a fluorescent protein and an enzyme can be re-constituted on SBA-NiO. Strikingly, we observed that a fragment of luciferase (in the absence of its complementary partner), when immobilized on SBA-NiO, exhibited the activity of the whole luciferase protein. To the best of our knowledge, this is the first example that only one half of an enzyme can exert the catalytic activity, albeit in spatially confined microenvironment. This is also the first example that site-specific interaction between proteins (or protein fragments) and material surface (NiO nanoparticles in mesopores) results in gain of catalytic function of an enzyme. Further exploration of the protein/SBA-NiO assembly by experimental and theoretical studies will reveal in greater details how spatial confinement in nano cavities alters the folding as well as the activity of proteins. Taken together, our discovery revealed an important aspect of basic protein biochemistry, the environmental factor. Aside from the chemical composition and three-dimensional organization, the physical environment represents another critical aspect that determines the activity and function of a protein.

## Supporting Information

S1 Fig Sequencing results of the plasmid encoding N1-mCherry (A), C1-mCherry (B), N2-mCherry (C) and C2-mCherry (D). Sequence highlighted in red encodes SSGSSGLVPRGSSGKLAAALE, as an additional peptide spacer inserted between the C fragment sequence and His-tag. Sequence highlighted in yellow encodes His-tag.
(DOCX)

S2 Fig Sequencing results of the plasmid encoding N-Luc (A) and C-Luc (B). Sequence highlighted in yellow encodes His-tag.
(DOC)
