# Supplementary material for: Functional Assembly of Protein Fragments Induced by Spatial Confinement
Source: PLoS One. 2015 Apr 15;10(4):e0122101. doi: 10.1371/journal.pone.0122101 (PMC4398348; doi:10.1371/journal.pone.0122101)
Supplement: S1 Fig — Sequence highlighted in red encodes SSGSSGLVPRGSSGKLAAALE, as an additional peptide spacer inserted between the C fragment sequence and His-tag. Sequence highlighted in yellow encodes His-tag. (DOCX) [file pone.0122101.s001.docx]

Electronic Supporting Information

**Functional Assembly of Protein Fragments Induced by Spatial Confinement**

Yongsheng Yu^1^, Jianpeng Wang^1^, Jiahui Liu^1^, Daishun Ling^2*^, Jiang Xia^1*^

^1^Department of Chemistry, Center of Novel Biomaterials, The Chinese University of Hong Kong, Shatin, Hong Kong, China

^2^Institute of Pharmaceutics, College of Pharmaceutical Sciences, Zhejiang University, 866 Yuhangtang Road, Hangzhou, Zhejiang 310058, China

(A) N1-mCherry

CATCATCATCATCATCACAAGCTTATGGTGAGCAAGGGCGAGGAGGATAACATGGCCATCATCAAGGAGTTCATGCGCTTCAAGGTGCACATGGAGGGCTCCGTGAACGGCCACGAGTTCGAGATCGAGGGCGAGGGCGAGGGCCGCCCCTACGAGGGCACCCAGACCGCCAAGCTGAAGGTGACCAAGGGTGGCCCCCTGCCCTTCGCCTGGGACATCCTGTCCCCTCAGTTCATGTACGGCTCCAAGGCCTACGTGAAGCACCCCGCCGACATCCCCGACTACTTGAAGCTGTCCTTCCCCGAGGGCTTCAAGTGGGAGCGCGTGATGAACTTCGAGGACGGCGGCGTGGTGACCGTGACCCAGGACTCCTCCCTGCAGGACGGCGAGTTCATCTACAAGGTGAAGCTGCGCGGCACCAACTTCCCCTCCGACGGCCCCGTAATGCAGAAGAAGACCATGGGCTGGGAGGCCTCCTCCGAGCGGATGTACCCCGAGGACTAA

(B) C1-mCherry

ATGGGCGCCCTGAAGGGCGAGATCAAGCAGAGGCTGAAGCTGAAGGACGGCGGCCACTACGACGCTGAGGTCAAGACCACCTACAAGGCCAAGAAGCCCGTGCAGCTGCCCGGCGCCTACAACGTCAACATCAAGTTGGACATCACCTCCCACAACGAGGACTACACCATCGTGGAACAGTACGAACGCGCCGAGGGCCGCCACTCCACCGGCGGCATGGACGAGCTGTACAAGAGCAGCGGCAGCAGCGGCCTGGTGCCGCGCGGCAGCAGCGGCAAGCTTGCGGCCGCACTCGAGCACCACCACCACCACCACTAA

(C) N2-mCherry

CATCATCATCATCATCACAAGCTTATGGTGAGCAAGGGCGAGGAGGATAACATGGCCATCATCAAGGAGTTCATGCGCTTCAAGGTGCACATGGAGGGCTCCGTGAACGGCCACGAGTTCGAGATCGAGGGCGAGGGCGAGGGCCGCCCCTACGAGGGCACCCAGACCGCCAAGCTGAAGGTGACCAAGGGTGGCCCCCTGCCCTTCGCCTGGGACATCCTGTCCCCTCAGTTCATGTACGGCTCCAAGGCCTACGTGAAGCACCCCGCCGACATCCCCGACTACTTGAAGCTGTCCTTCCCCGAGGGCTTCAAGTGGGAGCGCGTGATGAACTTCGAGGACGGCGGCGTGGTGACCGTGACCCAGGACTCCTCCCTGCAGGACGGCGAGTTCATCTACAAGGTGAAGCTGCGCGGCACCAACTTCCCCTCCTAA

(D) C2-mCherry

GACGGCCCCGTAATGCAGAAGAAGACCATGGGCTGGGAGGCCTCCTCCGAGCGGATGTACCCCGAGGACGGCGCCCTGAAGGGCGAGATCAAGCAGAGGCTGAAGCTGAAGGACGGCGGCCACTACGACGCTGAGGTCAAGACCACCTACAAGGCCAAGAAGCCCGTGCAGCTGCCCGGCGCCTACAACGTCAACATCAAGTTGGACATCACCTCCCACAACGAGGACTACACCATCGTGGAACAGTACGAACGCGCCGAGGGCCGCCACTCCACCGGCGGCATGGACGAGCTGTACAAGAGCAGCGGCAGCAGCGGCCTGGTGCCGCGCGGCAGCAGCGGCAAGCTTGCGGCCGCACTCGAGCACCACCACCACCACCACTAA

**S1 Fig. Sequencing results of the plasmid encoding N1-mCherry (A), C1-mCherry (B), N2-mCherry (C) and C2-mCherry (D).** Sequence highlighted in red encodes SSGSSGLVPRGSSGKLAAALE, as an additional peptide spacer inserted between the C fragment sequence and His-tag. Sequence highlighted in yellow encodes His-tag.
